# Navigating the path to corneal healing success and challenges: a comprehensive overview

**DOI:** 10.1038/s41433-025-03619-2

**Published:** 2025-02-12

**Authors:** Athar Shadmani, Albert Y. Wu

**Affiliations:** 1 Bascom Palmer Eye Institute, University of Miami, Naples, FL USA; 2 Omid Salmat Clinic, Firozabad, Shiraz University of Medical Sciences, Firozabad, Iran; 3 Department of Ophthalmology, Stanford University School of Medicine, Stanford, CA USA

**Keywords:** Mechanisms of disease, Cell migration

## Abstract

The cornea serves to protect the eye from external insults and refracts light to the retina. Maintaining ocular homeostasis requires constant epithelial renewal and an efficient healing process following injury. Corneal wound healing is a dynamic process involving several key cell populations and molecular pathways. Immediately after a large corneal epithelial injury involving limbal stem cells, conjunctival epithelial cells migrate toward the center of the wound guided by the newly formed electrical field (EF). Proliferation and transdifferentiation play a critical role in corneal epithelial regeneration. Corneal nerve endings migrate through the EF, connect with the migrating epithelial cells, and provide them with multiple growth factors. Finally, the migrated epithelial cells undergo differentiation, which is also regulated by corneal nerve endings. All these processes require energy and effective cellular cross-talk between different cell lines and extracellular matrix molecules. We provide an overview of the roles and interactions between corneal wound regeneration components that may help develop fascinating new targeted therapeutic strategies to enhance corneal wound healing with less injury-related corneal opacity and neovascularization.

## Introduction

The cornea is a transparent structure in the anterior part of the eye that plays a critical role in vision. Its transparency and curvature are critical for refracting and focusing light on the retina and initiating perceived vision.

Healthy corneal epithelial cells (ECs) predominantly receive their metabolic requirements from the tear film and glycogen stores within their cytoplasm [1]. This stored energy makes ECs independent of the corneal avascular environment [1, 2]. The polysaccharide glycogen undergoes conversion to glucose and is utilized through glycolysis. The released energy is utilized for cellular movement and proliferation [3, 4].

In contrast, the conjunctival ECs is characterized by the absence of intracellular glycogen vacuoles and the presence of goblet cells scattered between ECs [2]. These cells are dependent on the extracellular blood supply to meet their metabolic demands.

Corneal regeneration after injury is a complex process involving stromal re-epithelialization. The initial phase of regeneration includes cellular and subcellular reorganization to trigger the migration of ECs at the wound edge [3, 5]. The next phase is cell migration, which is independent of cell mitosis [6]. Other steps include cell proliferation, differentiation and eventually stratification to restore the original multicellular epithelial layer [5, 7, 8].

Failure of corneal wound healing is a significant clinical problem that results in corneal opacity, corneal neovascularization and low vision. This failure is due to corneal inflammatory, infectious or chemical and physical injuries, including an increasing number of refractive surgeries. We provide an overview of cells, molecules, and pathways involved in corneal wound healing and explain how they are connected to each other and affect regeneration outcomes. Understanding these pathways may help identify fascinating new targeted therapeutic strategies that will enhance corneal wound healing with less injury-related corneal opacity and neovascularization.

## Corneal regeneration

Following epithelial injury, highly regulated inflammatory reactions occur, leading to limbal stem cell proliferation and migration [9, 10] to cover the wound, at approximately a rate of 100 μm per hour (as evidenced in a rabbit cornea model) [11]. Epithelial healing concludes with the formation of adhesion structures anchoring the regenerated epithelium to the underlying stroma [12].

In the early phase of regeneration, the cornea is covered by one to two squamous cell layers with no goblet cells. Goblet cells appear at the limbal edge within the first to third weeks and then reach a uniform distribution across the entire cornea. Subsequently, during transdifferentiation, the conjunctival ECs evolve into corneal ECs, and the goblet cells recede from the centre toward the limbus. Transdifferentiation lags behind defect closure by 4 to 5 weeks [2, 13, 14].

**Fig. 1:**
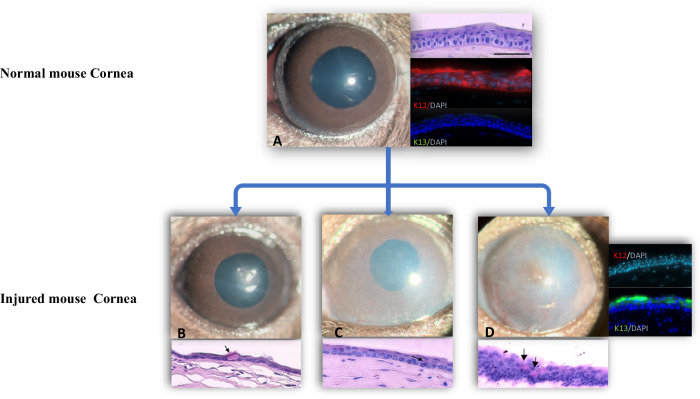
Healthy and injured mouse cornea. **A** Normal mouse cornea is a transparent tissue that consists of squamous cell epithelium with no goblet cells. It expresses corneal-specific antigen K12 on the corneal surface. K13, which is the conjunctival specific antigen is not expressed on the normal corneal surface. **B** Images of regenerated mouse cornea two weeks after Algerbrush injury. The corneal epithelium is regenerated, and goblet cells are visible on the PAS-stained epithelium (arrow). The thickness of regenerated epithelium is thin and atrophic. **C**, **D** Mouse cornea two weeks after severe NaOH injuriy with signs of stem cell deficiency and conjunctivalization, corneal opacity, presence of goblet cell (arrows) in PAS-stained sample. On the regenerated corneal surface K13 is expressed on the corneal surface and the K12 is absent. These are evidence of failure epithelial cells transdifferentiation to the corneal epithelial cells. The scale bar = 50 µm.

Two signs of successful conjunctival transdifferentiation to corneal ECs are the appearance of glycogen in the cytoplasm and K12, a corneal EC-specific antigen (Fig. 1) [15, 16].

Corneal nerve endings exert meticulous monitoring and control at each stage because of their close relationship with ECs. The progression of these stages is mediated by intricate electrochemical gradients and pathways.

## Factors regulating corneal regeneration

The most important factors that influence corneal epithelium healing after the injury can be divided into four major categories: 1. the corneal stem cell reservoir, 2. blood supply and initial inflammation, 3. corneal nerve endings, and 4. electrochemical gradients, extracellular matrix and cellular cross-talk.

### Stem cell reservoir

Stem cells are critical for corneal maintenance and regeneration. Under steady conditions, the limbus provides a physical and biochemical barrier that separates the cornea from the conjunctiva [17]. Stem cell activity in limbal niches is dependent on the proper limbal structure and guidance of nerve endings [18].

Limbal stem cells occasionally divide and undergo terminal differentiation and eventually desquamation to replace lost cells during corneal epithelial maintenance [19, 20]. However, in response to injury, the proliferative rate increases 8- to 9-fold in the limbus and approximately 2-fold in the peripheral and central regions [19, 21]. These cells give rise to many transient amplifying cells (TACs) with high migratory and proliferative capacity. Throughout the healing process, their properties are modulated as they undergo centripetal migration in response to changes in the extra cellular matrix (ECM), integrin receptors, growth factors and cytokines [22, 23]. The mitosis rate of cells returns to basal levels after 36-48 h in the limbus and after wound closure in the peripheral/central corneal region [21].

Notably, central corneal cells significantly contribute to the healing of small wounds, but in large wounds, limbal stem cells proliferate and migrate toward the wounded cornea. The main mechanisms involved in cell migration are an increasing population pressure gradient and basal cell migration from the limbus [1, 24, 25].

In patients with limbal stem cell deficiency (LSCD), corneal wound cause serious problems, such as delayed wound healing, stromal neovascularization and conjunctival cell ingrowth, which may cause corneal opacity and visual loss.

In LSCD patients, wound healing relies on conjunctival EC migration [26]. Conjunctival cells proliferate and migrate to the corneal surface to cover the bare stroma [1, 2, 27–30]. During transdifferentiation (metaplasia), conjunctival ECs transform into corneal ECs morphologically. If transdifferentiation fails, persistent inflammation and fibrovascular pannus result (Fig. 1) [8, 26, 31].

### Blood vessels and initial inflammation

Cellular metabolism depends on the energy provided to tissues and cells. Adenosine triphosphate (ATP) not only serves as a vital intracellular energy source but also functions as an important extracellular signalling molecule [32, 33].

ATP is an essential molecule for maintaining the cell membrane’s physiologic electrical gradient by providing energy for the Na^+^/K^+^ ATPase enzyme. ATP release within one minute after injury results in intracellular calcium mobilization upon purinergic receptor P2Y or P2X activation. This activation appears to be one of the earliest events in the healing process [34]. ATP enhances ECs migration and nerve sprouts through the corneal EF toward the wound centre (discussed in “Electrical Field“) [35].

Corneal injury and the healing process increase the energy demand, triggering blood vessel formation by increasing angiogenic factors such as angiogenin, hepatocyte growth factor (HGF) and vascular endothelial growth factor (VEGF) [36, 37]. Newly formed vessels meet regenerating cells’ high metabolic demand and drain cytokines from the injury site.

**Fig. 2:**
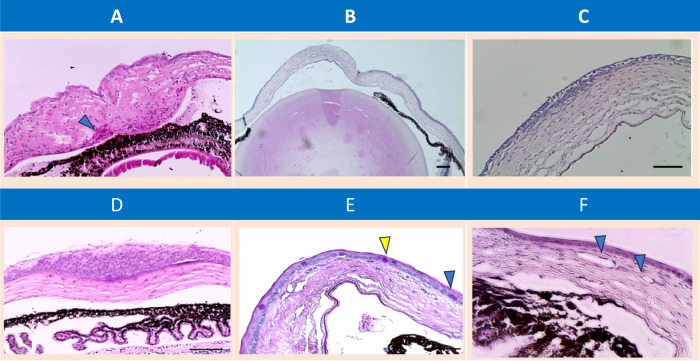
H&E and PAS staining of mouse corneas at different time points after injury. **A** Twenty-four hours after injury, RBCs (arrowhead), leucocytes and platelets are recruited to the corneal stroma. Iridocorneal adhesion enhances the migration of RBCs and inflammatory cells towards the corneal stroma (20x). **B** At four days after injury, stromal keratocytes are migrating from the re-epithelialized part of the cornea to the non-re-epithelialized centre of the cornea (10x). **C** Keratocytes accumulate at the junction of the newly formed epithelium and stroma, which is a sign of a close interaction between stromal cells and the regenerating epithelium after corneal wounding (20x). **D** At four days after injury, conjunctival epithelial cells accumulate at the limbus and slide over the cornea due to population pressure (40x). **E** The corneal surface is covered by atrophic epithelium with goblet cells scattered throughout the surface. The limbal epithelium has returned to its normal monocellular layer state 7 days after injury (blue arrowhead). **F** Failure of effective transdifferentiation of regenerated epithelial cells results in persistent inflammatory cells in the stroma and corneal NV at the deep stoma (arrowhead). Scale bar = 50 µm.

Damaged epithelial cells secrete the cytokine IL-1a, stored in ECs. Secreted IL-1a can cause increased corneal immune infiltration, initiating the inflammatory response [38]. Neutrophils, lymphocytes, platelets, and RBCs are recruited to the wounded cornea through the limbus and anterior chamber (Fig. 2A) [13, 39]. Platelet accumulation in the limbus and migration to the stroma and neutrophil migration into the wounded epithelium occurs through cell adhesion molecules such as P-selectin [40].

Initial inflammatory cells, especially platelets and neutrophils, play a significant role in the early increase in VEGF in the wounded cornea [41]. VEGF is essential not only for new blood vessel formation but also for corneal nerve regeneration [39, 42, 43]. Anti-VEGF administered systemically before injury or topically after injury markedly slows nerve regeneration [43].

Whether newly formed blood vessels undergo maturation or regression in the injured cornea is closely regulated by the corneal nerve endings.

Prolonged or excessive release of pro-inflammatory cytokines (e.g., IL-1, TNF-α) can result in chronic inflammation, which damages corneal tissues and contributes to haze or scarring.

Overactivation of matrix metalloproteinases (MMPs), such as MMP-9, can degrade the extracellular matrix, preventing proper healing and causing persistent epithelial defects that is visible in persistent corneal ulcers or autoimmune keratitis [44]. On the other hand, insufficient Inflammatory response impairs recruitment of immune cells, delaying the clearance of pathogens and debris, which can lead to non-healing wounds [45].

### Corneal nerve endings

The nervous system closely controls and monitors all cell migration and differentiation stages through abundant intracorneal nerve (ICN) endings [46]. ICNs contribute to the maintenance of corneal integrity.

The intracorneal nerve can be divided into sensory and autonomic nerve fibres (ANFs). Sensory nerve endings play a vital role in the maintenance of corneal integrity by secreting trophic neuropeptides such as substance P (SP), calcitonin gene-related peptide (CGRP), pituitary adenylate cyclase-activation polypeptide (PACAP), and vasoactive intestinal peptide (VIP) [47–49]. Corneal ANFs balance physiologic function through the sympathetic and parasympathetic nervous systems (SNS and PSNS, respectively). The PSNS primarily diminishes inflammation and promotes cell deviation, while the SNS induces increased inflammation and diminishes cell mitosis [50].

There is a close relationship between corneal ECs and ICN endings [51]. Establishing this close connection between regenerating ECs and ICN endings is critical for corneal epithelial cell transdifferentiation. Corneal nerve fibres and epithelial cells mutually support each other through bidirectional neurotrophic factor release, which is essential for robust ECs and nerve regeneration [43, 52–54].

**Fig. 3:**
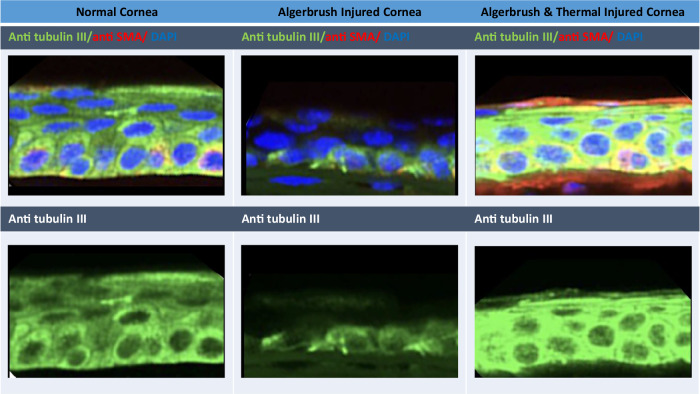
The thickness of the regenerated corneal epithelium is influenced by the thickness of the regenerated corneal nerve endings. The regenerated corneal epithelial thickness was less than normal in both the Algerbrush and the Algerbrush and thermally injured corneas. In the Algerbrush-injured group, which exhibited a thinner epithelium, βIII tubulin (a marker of corneal nerve endings) was expressed in only half of the epithelium thickness. However, in the Algerbrush and thermally injured cornea, which had a greater thickness of regenerated epithelium, the expression of anti-tubulin III was visible in the total height of the epithelium. Subepithelial α-SMA, a marker of corneal fibrosis, was more highly expressed in the Algerbrush and thermal injury group [16].

Newly formed regenerating ECs with more nerve connections receive more trophic neuropeptides which induces a thicker regenerated epithelium and enhances transdifferentiation (Fig. 3) [16]. This results in reduced inflammatory cytokine secretion and blood vessel regression (Fig. 6) [55].

At the same time, corneal ECs and keratocytes enhance corneal nerve survival and maturation by releasing neuropeptides, neurotrophins, and growth factors such as nerve growth factor (NGF), neurotrophin 3 (NT-3), neurotrophin 4/5 (NT-4), epidermal growth factor (EGF), brain-derived neurotrophic factor (BDNF), ciliary neurotrophic factor (CNTF), and glial cell derived neurotrophic factor (GDNF) [56, 57].

Impaired connection between corneal nerve and the new migrated EC delayed cell maturation and failure of transdifferentiation; therefore, these unmature ECs persistently secrete inflammatory cytokines that trigger angiogenesis, delayed epithelial healing, neuropathic pain, and persistent neurotrophic keratopathy (Fig. 6) [58, 59].

Newly formed epithelial cells and nerve endings also determine the fate of new formed vessels. The NGF improves perivascular innervation and sensitivity of new vessels to environmental mediators [59, 60]. The NGF guides new vessels to regress in regions where the corneal epithelium is healed with less metabolic demand, which results in secretion of fewer inflammatory factors. Conversely, NGF supports the persistence of new vessels in areas with undifferentiated conjunctival ECs with high metabolic demands [59, 61].

Corneal nerve damage during injury can impair wound healing through reduced production of neurotrophic factors like NGF This condition can be seen in neurotrophic keratopathy.

Consequences include delayed epithelial healing, neuropathic pain, and persistent neurotrophic keratopathy.

### Factors influencing cellular cross talk and intercellular connections

#### Growth factors/cytokines in epithelial wound healing

The epithelial growth factor (EGF) family, which is involved in epithelial wound healing, includes EGF, transforming growth factor-α (TGF-α), and heparin-binding EGF-like growth factor (HB-EGF) [62, 63]. These factors act via specific cell-surface tyrosine kinase, such as epithelial growth factor receptors (EGFR) [64, 65]. HB-EGF is a potent mitogen and chemoattractant for many cell types, including keratinocytes and epithelial cells [66].

Keratinocyte growth factor (KGF) and HGF are produced by stromal keratocytes, and their receptors are expressed in the epithelium [67, 68]. These findings clarify how stromal keratocytes support regenerating epithelial cells during wound healing (Fig. 2 B, C) [62].

Insulin-like growth factor-I (IGF-I) and insulin are multifunctional regulatory peptides that regulate cell proliferation, differentiation, and survival [69]. Their receptors are expressed by both ECs and fibroblasts in human corneas [69]. Transforming growth factor-ß (TGF-ß) inhibits EC proliferation and significantly stimulates corneal stromal fibroblast proliferation [70]. The different types of GFs and their receptors and pathways are illustrated in Table 1.

**Table 1:** Different growth factors involved in corneal regeneration.

Growth Factor	Producing cells	Target cells	Receptor	Pathway	Function		Other
					Migration	Proliferation	
EGF Family (EGF/ HB-EGF/ TGF- α)	Platelets/ Macrophages/ Fibroblasts	Epithelial cells	EGFR/erbB1/HER1 erbB2/HER2/neu erbB3/HER3 erbB4/HER4	Tyrosine Kinase NF-κB pathway histone deacetylase 6 PI3K–Akt and ERK PAX6 downregulation	+	+	Inhibit expression of K3 (differentiation marker)
KGF	Fibroblasts/ Keratocytes/ Endothelial cells	Epithelial cells	FGFR2b isoforms	Ras-MAPK PI3K/p70 S6		+	
HGF	Fibroblasts/ Keratocytes	Epithelial cells	c-Met (tyrosine activity Grb2/Sos	Ras-MAPK PI3K/AKT P70 S6K Pr kinase C	+	+	potent mitogen chemo-attractant
IGF-1	Epithelial cells	Epithelial cells	IGF-1 receptor	PI3K-Akt	+	+	increase chemotaxis in corneal fibroblast
Insulin	Beta cells in pancreas	Epithelial cells/Keratocytes/Endothelial cells	EGFR	ERK and PI3K		+	Maintain cell phenotype, prevent proteoglycan degeneration
TGF-ß	Epithelial cells	Epithelial cells/Keratocytes/ Endothelial cells	RI and RII	p38 MAPK	+	+	Extracellular matrix synthesis, Angiogenesis, increase keratocyte proliferation and myofibroblast differentiation

*EGF* epithelial growth factor, *EGFR* epithelial growth factor receptor, IGF-I, Insulin-like growth factor-I, KGF keratinocyte growth factor, TGF-α transforming growth factor α, *TGF-β* transforming growth factor β, *HB-EG* heparin-binding EGF, *HGF* hepatocyte growth factor.

#### Integrity of the epithelial cell membrane and the presence of receptors

The availability of adequate energy enables regenerating ECs to produce the necessary receptors and intracellular signalling pathways to promote proliferation and migration and inhibit cell apoptosis in regenerated cornea [71, 72].

In the early stages of epithelial wound healing, EGFR signalling activates the NF-κB pathway, which leads to transcriptional repressor CTCF activation and PAX6 downregulation. EGFR tyrosine kinase activity leads to the activation of the phosphatidylinositol-3-kinase (PI3K)-Akt axis and extracellular regulated kinase (ERK) [73, 74]. The outcome is enhanced EC migration and proliferation and inhibited expression of the differentiation-related corneal epithelial marker keratin 3 [75, 76].

Enhanced intracellular signalling facilitates EC healing and mitochondrial function recovery [71]. see section “More effective approaches for enhancing corneal wound healing” for more information.

#### Electrical Field

**Fig. 4:**
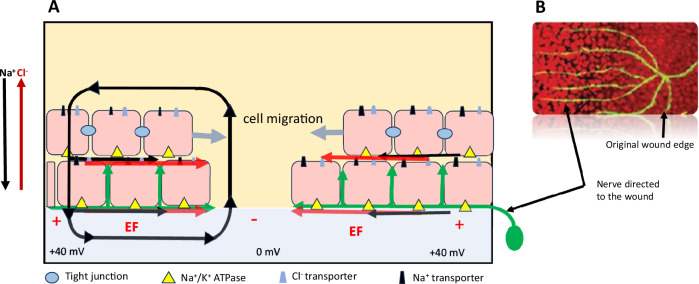
Effect of EF on the migration of corneal epithelial cells and nerve sprouts to the wound. **A** Tight junctions in the upper layers of the intact epithelium seal neighbouring cells together. The apical domain of epithelial cells is enriched in Na^+^ channels (black) and Cl^-^ transporters (blue), whereas the basolateral domain contains Na^+^/K^+^ ATPases (yellow); this enrichment results in the net movement of Na^+^ into the stromal layer and Cl^-^ into the tear fluid (arrows). The separation of ions with different charges creates a measurable transepithelial potential (TEP) difference, with the stroma being more positive than the tear fluid. Upon injury, the TEP instantaneously collapses to zero at the wound centre, but it remains at ∼40 mV distally. This voltage gradient establishes an EF (red arrows) in the corneal tissues that has a vector parallel to the epithelial surface and the wound centre. The black arrows indicate positive ion flow and current through tissues and the return path through the tear fluid. **B** In addition to directing epithelial cells, the EF steers neuronal growth to the injured cathodal wound centre (green arrows). Establishing the EF after injury is a critical factor in corneal regeneration.

**Fig. 5:**
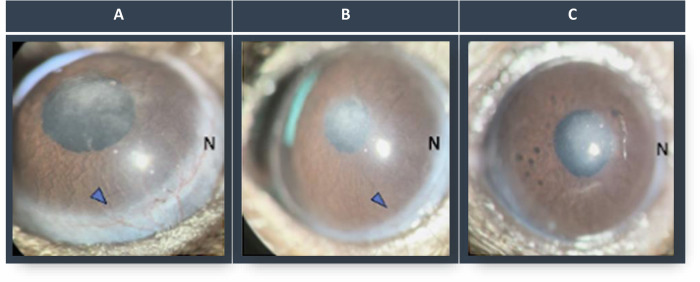
Corneal neovascularization at different time points after Algerbrush injury. **A** Seven days after injury, the new vessels are prominent in the limbal area that extends to the corneal surface (arrowhead). **B** Ten days after injury, the cornea shows thin limbal vessels with shorter extensions toward the corneal surface. **C** Two weeks after injury and successful transdifferentiation, the new corneal vessels regress, and the inflammation subsides, resulting in a clear cornea at the periphery. In the corneal centre, corneal opacity is still present, which is a sign of failed transdifferentiation of the regenerated corneal epithelium.

The endogenous EF is generated by ion channels, pumps, and electrical synapses (gap junctions) on the plasma membrane. Tight junctions between corneal ECs and the specific distribution of Na^+^ channels, Na^+^/K^+^ ATPases and Cl^-^ transporters result in a measurable transepithelial potential (TEP) difference of almost 40 mV (Fig. 4). Upon injury, the TEP instantaneously collapses to zero at the wound centre, but it remains at almost 40 mV distally, where ion transport is unaffected [77]. This voltage gradient establishes an EF in corneal tissues that has a vector parallel to the epithelial surface with the wound centre as the cathode (Fig. 5) [78]. This EF directs cell migration and neuronal growth during re-epithelialization by steering cells toward the centre of the cathodal wound [79]. Nerve bundles grow at right angles directly toward a wound edge in the mammalian cornea [80], and more nerves sprout when the EF is increased. In contrast, collapsing the EF permits only sparse nerve sprouting, which results in a loss of trophic interaction between epithelial cells and nerve endings [81].

ATP signalling plays a role in the electric field-guided corneal EC migration. In addition to providing energy for transporter and enzyme synthesis, ATP enhances cell migration in the EF and sensitizes cells to very low electric field levels (10-30 mV/mm) [35]. Cells stimulated with extracellular ATP migrated with significantly increased speed and directedness. Moreover, pharmacological ATPase inhibition enhanced cell migration in the EF. These findings support the necessity of ATP for successful regeneration [35].

Overall, the significance of crosstalk between several growth factors through the signalling pathway transactivation and between growth factors and extracellular mediators in corneal wound healing has been shown. The EF enhances and improves crosstalk, especially between charged proteins, and facilitates the complex process of epithelial wound healing.

## Corneal regeneration outcome

As the healing process progresses and the ECs cover the entire corneal surface, the corneal and conjunctival cell mitotic rate decreases. Successful regeneration helps cells produce ATP to maintain the ionic gradients of the plasma membrane and cell function, which results in a decrease in the secretion of inflammatory cytokines such as b-FGF and VEGF. Subsequently, new vessels and goblet cells regress, and TGF-β release decreases [13, 60, 82]. Along with the regeneration of ECs and the basement membrane, TGF-β leakage into the stroma is reduced, resulting in myofibroblast apoptosis. This process leads to the resolution of fibrosis and corneal opacity reduction over time (Fig. 5) [16, 83–85].

Insufficient levels of one or more factors result in regeneration failure. Persistent TGF-β signalling amplification due to continuous inflammation or induces excessive differentiation of keratocytes into myofibroblasts. These myofibroblasts produce abnormal extracellular matrix (e.g., fibronectin and type III collagen) and lead to scarring or haze [86].

Damage to LSCs induces long-term inflammation and the recruitment of macrophages and other inflammatory cells [87, 88].

Inadequate nerve endings due to infection, inflammation and trauma can directly destroy the maintenance of the corneal epithelium and cause neurotrophic corneal ulcers [89]. Previous studies have shown that after corneal injury, the temporal part of the cornea, which has more abundant nerve endings, has a faster recovery and less NV and opacity. In contrast, the nasal part of the cornea, which has a lower density of nerve endings, is more prone to developing NV and opacity after injury [13, 90].

Wound healing in individuals with sensory neuropathies and diabetes is slow due to inadequate sensory nerve endings and poor neurotrophic interactions between epithelial cells and sensory nerve sprouts [53, 91].

**Fig. 6:**
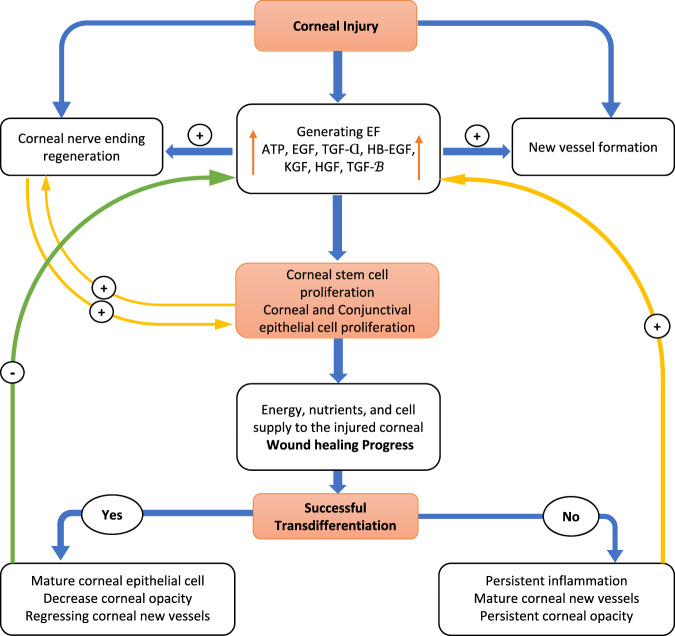
Corneal injury and factors influencing healing process outcomes. Corneal injuries may result in either successful healing with a clear cornea or progress toward a persistent epithelial defect accompanied by inflammation and the formation of mature new vessels. ATP Adenosine triphosphate, EF electrical field, EGF epithelial growth factor, EGFR epithelial growth factor receptor, KGF keratinocyte growth factor, TGF-α transforming growth factor α, TGF-β transforming growth factor β, HB-EGF heparin-binding EGF, HGF hepatocyte growth factor.

Inadequate energy and ATP availability result in the loss of EC plasma membrane integrity, function and electrical gradients. This loss results in failure of the growth and establishment of close connections and interactions with nerve endings, inadequate transdifferentiation, persistent inflammation, and fibrosis (Fig. 6) [1, 92, 93].

## More effective approaches for enhancing corneal wound healing

Given the various factors contributing to the corneal wound healing process, addressing injured and inflamed tissue with the necessary material can enhance the healing process. Extensive inflammation and ocular surface dryness are two major contributing conditions that should be addressed carefully.

Lubricating the injured cornea can be achieved through various methods, including the use of synthetic artificial tears, blood-derived products [94], and ciliary nerve electrostimulation [95].

In the context of severe inflammation use of anti-inflammatory drugs, including corticosteroids and non-steroidal anti-inflammatory drugs (NSAIDs), creates a microenvironment that is more favourable for nerve regeneration [96]. Doxycycline, which has anti collagenolytic activity can facilitate nerve repair by reducing excessive inflammation [97].

Recombinant nerve growth factors and cytokines have revolutionized the management of neurotrophic corneal ulcers. Oxervate® (cenegermin) is an FDA-approved recombinant human nerve growth factor (rhNGF) which promotes corneal nerve healing and epithelial repair. It significantly improves nerve regeneration in neurotrophic keratitis, herpetic and diabetic keratopathy where nerve damage prevents corneal healing [98].

In stem cell-deficient and chemically injured eyes, stem cell transplantation enhances corneal repair by improving CN density and function. Stem cells can directly differentiate into corneal tissues, including the corneal stromal cells, epithelial cells, and neurons. Another key supportive mechanism of stem cells in tissue regeneration is through their paracrine effects. They release various growth factors in the form of exosomes or secretomes which induce corneal wound healing and reduce fibrosis [99–101].

In extensive corneal injuries with large stromal and epithelial defects, microenviromental support is essential to enhance corneal nerve and epithelial cell regeneration. Recombinant collagen and hydrogel construct like CACICOL20® [102] and natural amniotic membrane products like Prokera® [103] are examples of structural support.

Stimulating chronic wounds with low-voltage electrical currents has the potential to enhance axonal migration and stimulate nerve endings by facilitating the development of a more efficient EF in the injured area [104, 105].

There are new experimental treatments that target specific pathways. Aquaporin-5 Modulators facilitate corneal epithelial wound healing and nerve regeneration by reactivating the Akt signalling pathway, aiding in quicker and more effective tissue repair [106].

To improve energy supply, Antioxidant Inflammation Modulators (AIMs) such as RTA 408 have been investigated. These agents activate the Nrf2 pathway, which is essential to produce cytoprotective molecules and mitochondria. This approach addresses oxidative stress and reduces free radicals and subsequent inflammation, during corneal repair especially in degenerative conditions [107, 108].

The future of corneal regeneration may focus on innovative biological treatments that address the energy demands of regenerating tissue, such as mitochondrial transplantation or ATP supplementation an emerging and promising field in ophthalmology.

## Conclusion

Corneal wound healing is a multifactorial process. Corneal regeneration success or failure is influenced by various factors. A lack of any factor results in the malfunction of pathways and cells influenced by that factor and causes insufficient regeneration. A clear understanding of these factors and their interactions can help us to develop more effective prevention and treatment modalities.
